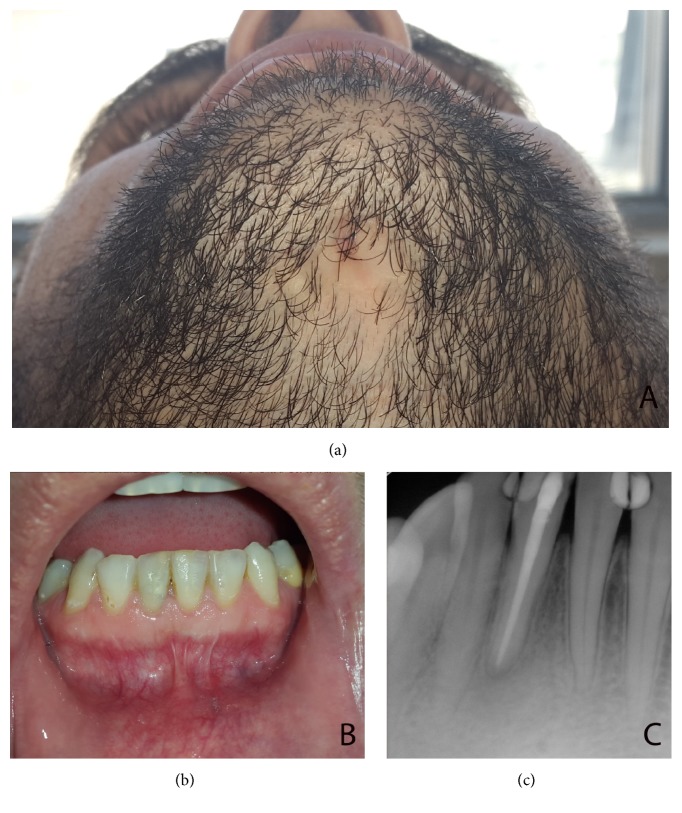# Uncommon Odontogenic Orocutaneous Fistula of the Jaw Treated with Platelet-Rich Fibrin

**DOI:** 10.1155/2017/7174217

**Published:** 2017-03-26

**Authors:** Kani Bilginaylar

**Affiliations:** Department of Oral and Maxillofacial Surgery, Near East University Faculty of Dentistry, Nicosia, Northern Cyprus, Mersin 10, Turkey

## Abstract

Orocutaneous fistula (OCF) of dental origin is a relatively rare condition and continues to be a challenging diagnosis. Misdiagnosis of OCF usually leads to unnecessary and noneffective treatment. A 21-year-old male referred with a complaint of a lesion on the chin which was misdiagnosed as a carbuncle (lesion of nonodontogenic origin) by a physician. After radiological examination, there was a lesion around the apical region of right central incisor. These findings indicated a sinus tract associated with dental origin. After root canal treatment, apical surgery was performed and platelet-rich fibrin (PRF) was administered to the cavity of the lesion as a gel form to improve healing and also used as a membrane form to cut off the relation between infected area and the skin. All procedures were performed intraorally; no extraoral intervention was performed. Three months later, clinical and radiological examination showed total healing without scar formation. The key to successful treatment of OCF is accurate diagnosis. Additionally, the use of PRF after surgical interventions is an effective and innovative therapy to improve healing.

## 1. Introduction

Orocutaneous fistula (OCF) of dental origin is a relatively rare condition and continues to be a challenging diagnosis [1, 2]. Frequently, the possibility of an odontogenic origin is overlooked so that patients prefer to refer to physicians for treatment of the cutaneous lesion, because most of the patients do not experience any symptoms associated with teeth [3]. Many patients can also show similar conditions in specific clinical anomalies such as epidermal cyst, furuncle, carbuncle, foreign body reaction, osteomyelitis, bisphosphonate-associated osteonecrosis, pyogenic granuloma, salivary gland fistula, thyroglossal tract fistula, branchial cleft fistula, actinomycosis, basal cell, and squamous cell carcinoma [1–4]. In this instance, physicians may not appreciate the chronic dental infection as the source of a draining sinus tract, and therefore, treatment may involve therapies directed at nonodontogenic diagnosis such as multiple antibiotic regimens, multiple surgical excisions, multiple biopsies, and radiotherapy, all of which failed [1–6]. Right diagnosis is required to prevent diagnostic dilemma and successful treatment of OCF. Therefore, dental aetiology should be considered for a right diagnosis if there is a draining lesion in the facial area.

This case includes a cutaneous odontogenic sinus tract to the chin that was misdiagnosed by a physician. However, complete healing following dental treatment was presented.

## 2. Case Report

**Figure 1:**
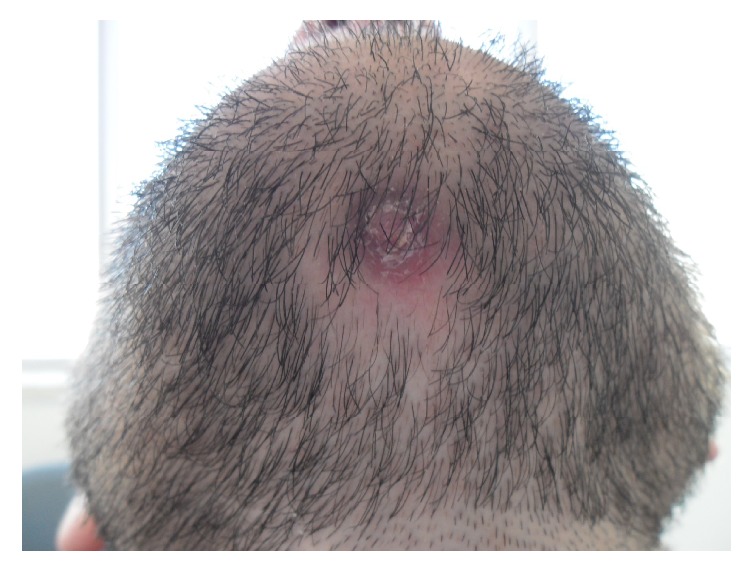


21-year-old male patient referred to department of oral and maxillofacial surgery with a complaint of drainage from a cutaneous lesion which was approximately 2 cm diameter under his chin (Figure 1). According to the history of the patient, he had first become aware of the lesion 6 months before contacting with the author. Initially, the lesion was diagnosed as a carbuncle by the physician and had been treated with combine systematic antibiotics (1 gr amoxicillin and clavulanate every 12 hours and 150 mg clindamycin every 6 hours for 7 days). After using these medicines the patient reported that for a while drainage was stopped and the lesion decreased in size. Since there was repeated occurrence (the lesion gradually increased and drainage started) of the lesion, he referred to our dental clinic for an alternative solution.

**Figure 2:**
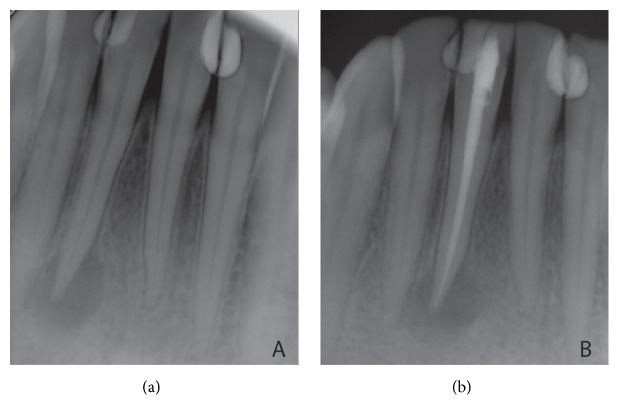


**Figure 3:**
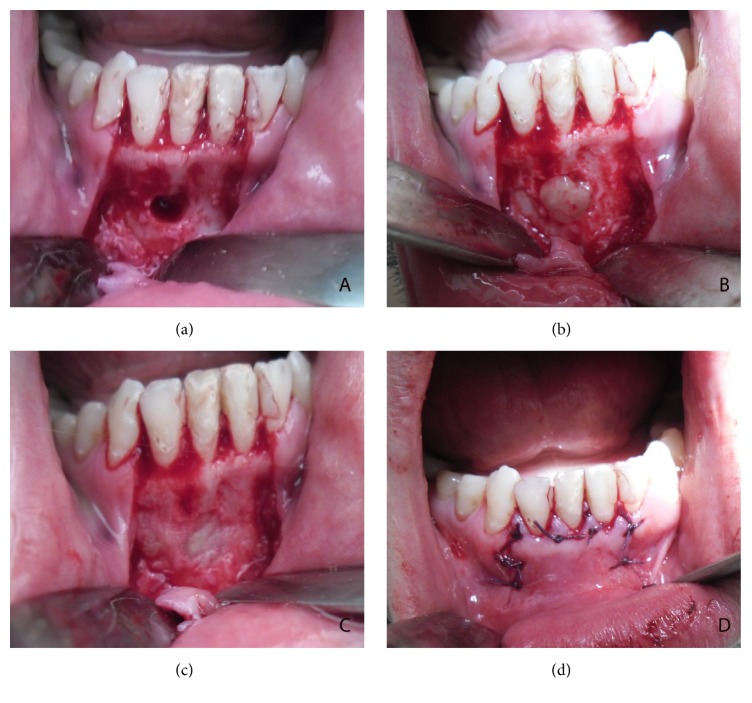


Extraoral examination revealed that there was a cutaneous lesion on his chin (Figure 1). Intraorally, although he had class III restorations on his mandibular anterior teeth (mesial sides of 32 and 42 and distal sides of 31 and 41), the teeth and periodontal tissues at anterior region of mandible seemed healthy. Furthermore, there was no pain either palpation or percussion. Dental ethology of sinus tract of odontogenic origin can be confirmed with the use of gutta-percha or similar radiopaque material but since the patient did not accept any extraoral interventions an intraoral periapical radiograph was taken without gutta-percha and it showed radiolucency around lower right central incisor which was the only reason that can cause cutaneous lesion. Radiolucency indicated inflammation of pulp that causes chronic apical periodontitis (Figure 2(a)). The treatment started with root canal treatment (Figure 2(b)). 2 days after the filling of the root canal, apical resection was performed and platelet-rich fibrin (PRF) was administered into the bone cavity and a PRF membrane was used to close the site as well. Following soft tissue closure with 3–0 silk sutures (Figure 3), patient was instructed to take amoxicillin (1000 mg) 3 times per day for five days and to use antiseptic (povidone-iodine 7.5%) mouthwash 3 times per day for seven days. Flurbiprofen (100 mg) was also prescribed postoperatively to be taken as required. Sutures were removed after seven days and the wound healed uneventfully.

In this presentation, no sinus excision was performed extraorally. At the third month follow-up, radiographical, intraoral, and extraoral examinations revealed complete healing of bone at periapical lesion area and spontaneous healing of orocutaneous fistula. Additionally, during the healing period, his old restorations on lower anterior incisors were renewed as shown in Figure 4.

*Preparation of PRF*. A blood sample of patient was taken directly into 10 mL glass-coated plastic tube which was not containing anticoagulant and immediately centrifuged (Elektro-mag M415P) at 3000 rpm for 10 min, approximately 10 min before the surgery. The platelet-poor plasma that accumulated at the top of the tubes was discarded. PRF was dissected approximately 2 mm below its contact point with the red corpuscles situated beneath, to include any remaining platelets that may have localized below the junction between the PRF and red corpuscles [7].

## 3. Discussion

A cutaneous fistula of dental origin is a pathway that begins at the apex of an infected tooth or of infected region of the jaw through the alveolar bone and drains infected materials (pus) through the skin [1, 2]. Treatment of this situation starts with an accurate diagnosis because there are many differential diagnoses. After extraoral, intraoral and radiological examination if the source of this lesion is from dental origin, the main approach should be to cut off communication between the infected area and the skin (to eliminate the source of infection). Intraorally, this can be done by nonsurgical conservative endodontic therapy and endodontic treatment followed by apical resection or extraction with curettage and surgical removal of sinus tract extraorally.

In the literature, many treatment modalities have been shown. According to Sammut et al. [1] in the first case, only root treatment was done; in second case, apical surgery and retrograde root filling were done; in third, fourth, and fifth cases, teeth associated with sinus tracts were extracted and healing was observed in all cases. Brown et al. [2] reported that sinus tracts related to teeth were extracted in three cases and lesions were healed uneventfully similar to that observed by Sato et al. [8]. According to Janev and Redzep, [3] after root canal treatment, apical resection and extraoral excision were performed and total recovery was observed. Kumar et al. [4] reported that sinus tracts of dental origin completely resolved only with conservative endodontic treatment similar to that observed by Tidwell et al. [5], Pasternak-Júnior et al. [9], and Tian et al. [10]. According to Giménez-García et al. [11] in the first case, extraction of the remains of several roots and extraoral excision was performed; in the second case, it was too late for endodontic treatment; therefore, the tooth with cyst was extracted. As a result of treatments they observed healed dental sinus. Gupta et al. [12] performed excision of sinus tracts, with extraction or after root canal treatment, Mishra and Khan [13] reported that a surgical excision of extraoral sinus tract was done followed by root canal treatment. In summary, all successful treatments of OCF include eliminating the source of infection and cuting off the pathway between the skin and infected area.

Although platelet-rich plasma (PRP, first generation of platelet concentrate) has been widely used in regenerative medicine, its preparation protocol is relatively complex and not standardized between laboratories. Therefore, its clinical outcomes have often varied significantly among individual clinical research groups [14]. To overcome this disadvantage, French Doctor Choukroun developed PRF (second generation of platelet concentrate) by modifying the process of PRP preparation [15]. There are some main differences between PRP and PRF. PRF can be prepared solely through the activation of an endogenous coagulation process without the aid of animal-derived coagulants and PRP needs 2-step centrifugation but PRF needs 1-step centrifugation which makes preparation of PRF easier than PRP. In addition, the final product of PRP can be used in liquid form but PRF can be used in gel and membrane form because of its fibrin matrix. Furthermore, physician-friendly handling and operator-friendly preparation procedures are also advantages of PRF when used in a clinical setting [14–16].

PRF is autologous fibrin gel that includes inflammation and healing mediators. Growth factors (Transforming Growth Factor (TGF-*β*1), Platelet-Derived Growth Factor (PDGF-*ββ*), Insulin-Like Growth Factor 1, and Vascular Endothelial Growth Factor (VEGF)), leukocytic cells, and their cytokines (tumor necrosis factor *α*, interleukin-1*β* [IL-1*β*], IL-6, and IL-4) are enmeshed within the PRF fibrin matrix. PRF has been used in wound healing, periodontal healing, angiogenesis, and bone augmentation with promising results. Choukroun et al. showed a cystic cavity filled with PRF and bone healed in 2 months instead of 6 to 12 months because PRF matrix is able to more efficiently direct stem cells harvesting and accelerates healing. Moreover, using of PRF as a membrane also includes all the molecular and cellular elements permitting optimal healing because the fibrin matrix carries all the favourable constituents present in a blood sample [7, 14, 16].

In the present case, 2 days after root canal treatment periapical surgery was done and PRF was applied to the resection cavity as a gel formation to accelerate healing and membrane version was used to cut off the pathway between alveolar bone and sinus tract. As a result of this treatment modality no scar formation occurred at the chin and spontaneous healing of sinus tract was observed.

## 4. Conclusion

A case of OCF is reported which presented as a diagnostic dilemma which completely resolved with the valuable help of PRF after root canal treatment and apical resection without any extraoral approach. All fistulas around the face and neck must be considered of odontogenic origin because accurate diagnosis of OCF brings success of treatment and prevents diagnostic dilemma. Additionally, the use of PRF after surgical interventions is an effective and innovative therapy to improve healing.

## Figures and Tables

**Figure 4:**